# An integrative piRNA analysis of mouse gametes and zygotes reveals new potential origins and gene regulatory roles

**DOI:** 10.1038/s41598-018-31032-1

**Published:** 2018-08-27

**Authors:** Eduardo Larriba, Jesús del Mazo

**Affiliations:** Centro de Investigaciones Biológicas C.I.B. (CSIC). Department of Cellular and Molecular Biology, Ramiro de Maeztu, 9, 28040 Madrid, Spain

## Abstract

Piwi-interacting RNAs (piRNAs) are a subclass of the small non-coding RNAs (sncRNAs). Their main reported function was to exert control over transposable elements (TEs) in mammalian germline. In this study undertaking a deeper bioinformatics analysis of piRNAs present in mouse oocytes, sperm cells and zygotes, we first elaborated a new piRNA database based on sequences identified as piRNAs by immunoprecipitation with PIWI proteins. Our bioinformatics analysis revealed that, at least in gametes and zygotes, piRNAs could encompass multifunctional cell-dependent regulatory molecules. Indeed, genome analysis of the piRNA mapping density (reads/kb) evidenced in all samples an enrichment of intron-derived piRNAs. Further, piRNA population was classified into sequences not associated to TEs or repeats (NRapiRNAs) and associated to repetitive genome elements (RapiRNAs). In oocytes most of the NRapiRNAs mapped to the 5′UTRs of coding mRNAs, while higher proportion of NRapiRNAs was detected in sperm cells associated to the 3′UTRs of mRNAs. This piRNA complementarity to mRNA UTRs suggests key post-transcriptional regulatory roles over mRNAs such as those encoding MHC genes. In addition, a striking association of RapiRNA with long non-coding RNAs (lncRNAs) was identified. piRNAs associated with relevant lncRNAs such as: *Rab26os* and *GAS5* and key mRNAs, were particularly assessed.

## Introduction

Non-coding RNAs (ncRNAs) play key roles as negative post-transcriptional gene regulators^1^ taking part in normal and pathological processes of cell differentiation and development^2^, which include germ cells and the reproductive systems^3^. Mutations and the deregulated expression of ncRNAs have been associated to diverse pathologies^4,5^. Small non-coding RNAs (sncRNAs) (of about 18–35 nucleotides), include the microRNAs (miRNAs), the Piwi-interacting RNAs (piRNAs) and endogenous-small interfering RNAs (endo-siRNAs), as well as other classes of small noncoding RNAs derived from tRNAs or rRNAs, in addition to the small nucleolar RNAs (snoRNAs)^1,6^. Other ncRNA types (>200 nt) are considered long non-coding RNAs (lncRNAs) implicated at different levels in gene expression regulation, including mechanisms of chromatin modification and genomic imprinting^7,8^.

Interestingly, long intergenic non-coding RNAs (lincRNAs) contain the highest percentage of transposon-derived sequences (TEs), suggesting an evolutionary participation in function gain^9^. Recently, a network model has been proposed which involves TEs and lncRNAs in DNA methylation regulation and embryonic stem cell reprogramming during embryogenesis^10,11^. Recent studies have begun to explore the regulatory interactions among the diverse types of non-coding RNAs^12^.

piRNAs comprise the most abundant class of small non-coding RNA molecules expressed in animal cells. With an average size ranging 24–32 nt, piRNAs interacting with PIWI proteins, a subfamily of the Argonaute proteins, show 5′ sequences enriched with uridine and 2′ O-methyl modifications at the 3′ end^13,14^. piRNAs are mainly processed from single-stranded precursor transcripts expressed throughout of all genomic regions, yet the biogenesis sources and mechanics remain little known^15^. piRNAs were considered essential for germ cell maintenance due their TE regulating competence by post-transcriptional silencing and DNA methylation transcriptional regulation^13,14,16–18^. However, new roles for piRNAs have recently emerged beyond those of transposon repression. These roles have also been detected in somatic cells asides from gonads such as central nervous system, heart or in cancer development^19–23^. By means of small RNAs-seq analysis we have initially identified piRNAs and endo-siRNAs in mouse gametes and zygotes that surprisingly showed origins and potential targets not only associated to TEs, but also derived from other non-coding RNAs^6,24^.

Both long and small ncRNAs can also be interconnected throughout of their biogenesis. A recent example of these complex interactions has been reported identifying lncRNA *GAS5*, which harbours snoRNAs and generates a piRNA capable to induce specific gene expression activation^25^. Likewise, piRNAs derived from tRNA or tRNA-derived RNA fragments (tRFs)^26^ have disclosed a new system of gene regulation^27,28^. Recently, the ability of tRFs to interact with PIWI proteins in human somatic cells^21^ has also been detected.

Implementing new bioinformatics approaches, in this study we have integrated genomic origins of piRNAs with potential functions as complex and versatile post-transcriptional regulatory elements, which by association with other RNAs^29–31^ could provide a new gene regulation dimension of key biological scenarios. For instance in differentiating cells such as gametogenesis and fertilization. In particular, we have assessed the differences between piRNAs either associated or not with TEs and genomic repeat elements in three of the most highly differentiated cells: oocytes and spermatozoa, and zygotes.

## Results

### Construction of a global immunoprecipitation piRNA database and use to identify piRNAs of oocytes, zygotes and sperm cells

First, we generated a new piRNA database exclusively based on previously reported sequences identified by RNA-Seq after immunoprecipitation with three mouse PIWI proteins: MIWI, MILI and MIWI2^32^ (Table S1). The methodological approach to generate this non-redundant immunoprecipitation piRNA database (IPpiRNA-db) is described in the methods section. It contains a total of 16,033,825 piRNA sequences that range from 15 to 40 nt in length, with a 24–31nt length enrichment characteristic of piRNAs (Fig. S1A). The piRNA nucleotide distribution indicated that 64.5% of the IPpiRNA sequences presented a 5′ uridine residue (Fig. S1B) also considered a piRNA hallmark^13–15^.

We next applied to the IPpiRNA-db a deeper identification and integrative analysis of our piRNA populations derived from the small RNA-Seq libraries previously generated from the mouse zygotes and gametes^24^. First, sequences having 5 counts or less that were not considered as representative were removed from the raw data set (% of sequences with less of 5 counts in oocyte, zygote and sperm were 18%, 16% and 6,5%, respectively), thus increasing the analysed sequence confidence. Furthermore, it had almost no effect on the total number of reads present in the previously analysed sncRNA-Seq libraries (% of cleaning reads in oocyte, zygote and sperm were 92%, 91% and 77%, respectively). Using this new piRNA database 34,417,108 piRNA reads, 31,236,897 piRNA reads and 24,123,710 piRNA reads were identified in oocyte, zygote and sperm cells respectively in our libraries. These corresponded respectively to 68%, 63% and 85% of the total oocyte, zygote and sperm cell clean reads (Table 1). Consequently, the generation of this new tool allowed to detect 28%, 29% and 22% more oocyte, zygote and sperm cell piRNA reads respectively than previously detected^6,24^. With this approach we only considered piRNAs those sncRNAs bound to PIWI proteins.

**Table 1:** piRNA identification statistics using the piRNA IP database.

	Oocyte		Zygote		Sperm	
	Reads	Sequences	Reads	Sequences	Reads	Sequences
piRNAs identified using Ipdb	34,417,108 (68%)	232,718 (51%)	31,236,897 (63%)	240,680 (53%)	24,123,710 (85%)	107,704 (33%)
piRNAs identified using Ipdb map genome	31,865,111 (63%)	229,233 (50%)	30,100,524 (61%)	238,150 (52%)	16,139,503 (57%)	104,010(32%)
Total sncRNA-seq	50,561,193	457,580	49,555,453	454,901	28,519,791	324,746

Percentages in brackets were calculated in relation to total number of sequences present in the sncRNA libraries.

### Analysis of piRNA length and nucleotide sequences analysis with regard to TE and repeat regions

#### piRNA population classification

piRNAs have been described to be associated with repeated regions and TEs. However, piRNAs can also be associated with other genomic elements^29–31,33,34^. To assess piRNAs with TEs and other repeat region associations, we designed a specific bioinformatics pipeline (Fig. S2). *Bona fide* piRNAs identified in the IPpiRNA-db were mapped to the specific mouse genome sequences presenting TE and repeat sequences identified by Repeat Masker (see Methods section). Using this bioinformatics approach, we classified piRNAs into two populations based on either their association or not with repeats, including TEs: Repeat-associated piRNAs (RapiRNAs) and non-repeat associated piRNAs (NRapiRNAs). The RapiRNA population corresponded to 98% of the oocytes and zygote reads and 90% of the male gamete reads, therefore indicating that the NRapiRNAs were underrepresented (Table 2). Nonetheless, in the somatic cells with a lower piRNA level, the proportion of NRpiRNAs was overrepresented with respect to the number of RapiRNAs^19,34^, suggesting differential functions in the germ *versus* the somatic cell lines.

**Table 2:** Total number of sequences and reads of two piRNA populations classified according to their TE associations.

	Oocyte		Zygote		Sperm	
	Reads	Sequences	Reads	Sequences	Reads	Sequences
RapiRNAs (piRNAs mapping to transposons or repeat sequences)	31,075,878 (98%)	225,404 (98%)	29,484,672 (98%)	234,680 (99%)	14,653,248 (91%)	93,473 (90%)
NRapiRNAs (piRNA not mapping to transposons or repeat sequences)	789,233 (2%)	3,829 (2%)	615,852 (2%)	3,470 (1%)	1,486,255 (9%)	10,537 (10%)
piRNAs identified using Ipdb map Genome	31,865,111	229,233	30,100,524	238,150	16,139,503	104,010

Percentages in brackets were calculated in relation to total number of piRNAs identified using the piRNA IP database according to the RapiRNA and NRapiRNA populations.

#### Length sequence distributions of the piRNA populations

**Figure 1:**
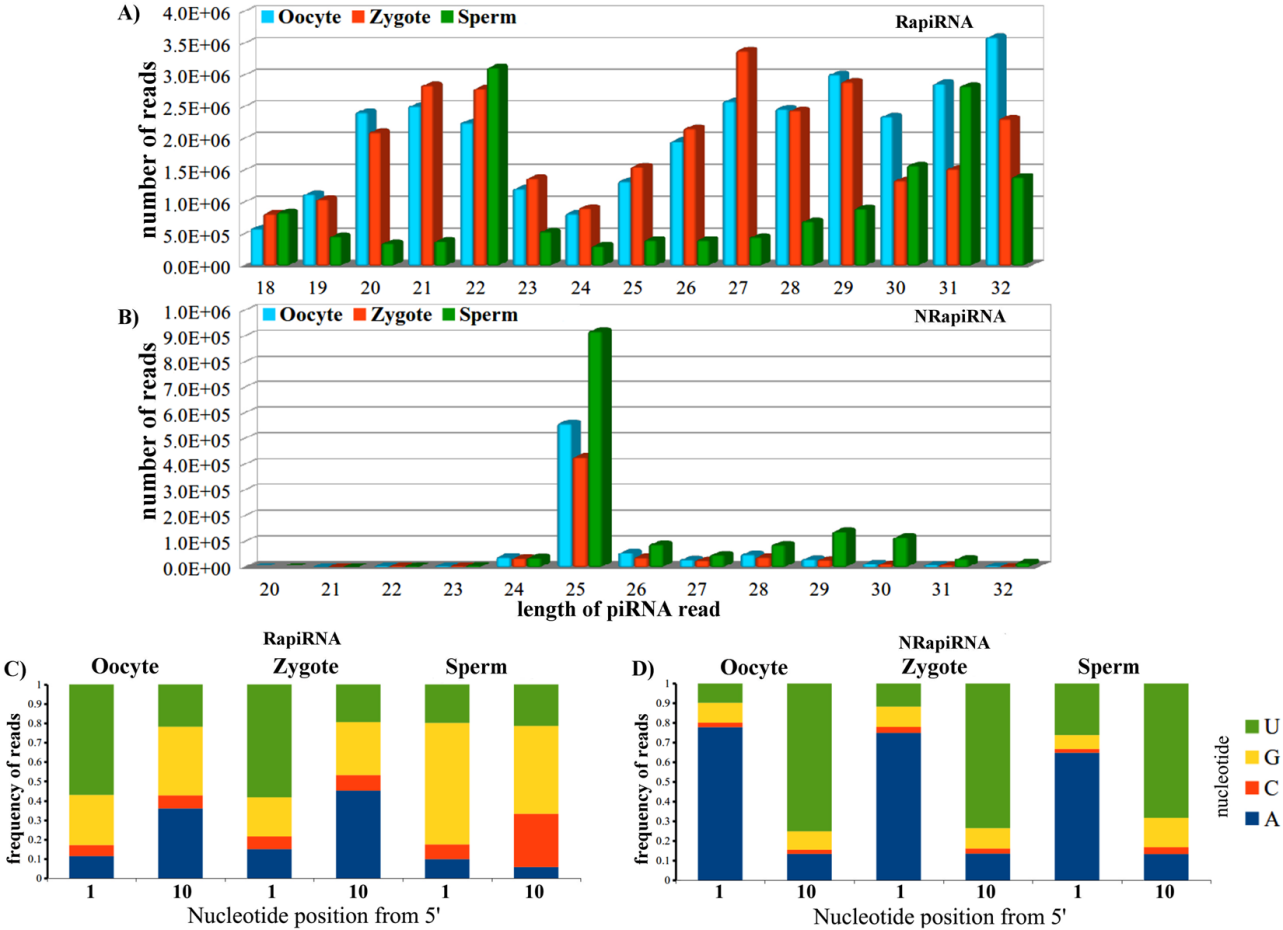
Lengths and nucleotide distributions of piRNAs associated and not associated with repeated elements. Panels A and B show the length distributions of piRNAs. C and D, the nucleotide frequencies of the first (1) and tenth (10) positions of the piRNA populations. Panels A and C are related to the piRNA population associated with TEs (RapiRNAs). Panels B and D are related to the piRNA population not mapping to TEs (NRapiRNAs).

The two piRNA populations here identified and classified displayed different features with respect to length and sequence (Fig. 1). Analysis of the length of the sequences of the RapiRNA population evidenced a bimodal distribution with 20–22 nt long sequences associated to rasRNAs (repeats associated RNAs) and involved in germline TE defence^1^, together with sequences containing 27 to 32 nt with a length closer to that considered as a canonical piRNA size (Fig. 1A). Interestingly, Kabayama and collaborators found in mouse oocytes sequences of 21–23 nt suggesting to be generated by piRNA pathway components in a non-canonical way^35^.

Globally, the oocyte and zygote samples presented a higher number of RapiRNA compared to sperm cells (Fig. 1A). In contrast, the NRapiRNAs displayed a defined peak at 25 nt with respect to all of cells types (Fig. 1B), exactly the opposite distribution of that observed for the RapiRNAs. These different piRNA population sizes have been associated with the selective interactions of the murine PIWI proteins in accordance with piRNA length sizes.

#### Nucleotide compositions of the piRNA population

A characteristic of the functional piRNAs that participate in the germ cell protection against TE propagation consists of the amplification mechanism termed ping-pong cycle^16,36^. Primary piRNAs from piRNA precursors display a bias for 5′ uridine (1U). Primary piRNAs associated to MILI/MIWI2 proteins to produce secondary piRNAs by sequence complementarity, generating a bias for adenine at position 10 (10A)^36,37^. We found differences in the relative nucleotide compositions at positions 1 and 10 nt between RapiRNA and NRapiRNA populations (Fig. 1C,D). In oocyte and zygote RapiRNAs 57–58% of the sequences presented uridine in the first 5′ position (1U) (Fig. 1C). Conversely, uridine was only detected in the first 5′ position in 21% of sperm cell RapiRNAs (Fig. 1C). In addition, the ping-pong cycle hallmark with adenine at position 10 was detected in oocytes, zygotes and sperm cells with frequency of 35%, 45% and 5.7% respectively (Fig. 1C). This RapiRNA population analysis suggests that in the spermatogenic differentiation piRNAs scarcely contemplate ping-pong amplification mechanisms (at least in the last period of spermiogenesis). Surprisingly, NRapiRNAs evidenced an inverse pattern. Adenine was the most frequent first position nucleotide with respect to 77%, 73% and 68% of the oocyte, zygote and sperm cell reads; being uridine the most frequent position 10 nucleotide (Fig. 1D).

Length distributions and nucleotide compositions revealed two clearly differentiated piRNA classes. The sequence patterns attending positions 1 and 10 suggest differential processing and functions. RapiRNA and NRapiRNA could had functional differences relative to their primary RNA targets, with mobile elements preferentially for RapiRNAs while other types of RNAs for NRapiRNAs.

### piRNA distribution along chromosomes

**Figure 2:**
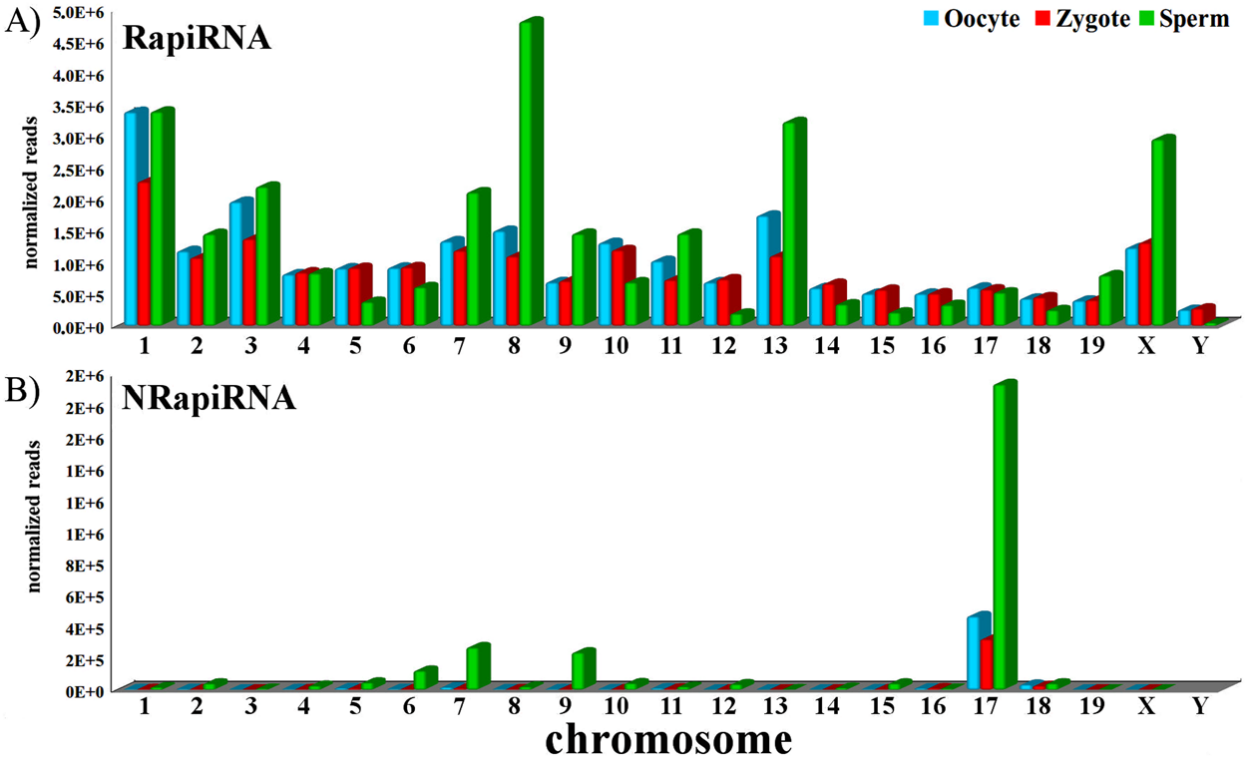
Mouse chromosome piRNA read distributions. Read counts of the three cell types were normalized using DESeq. RapiRNAs: Distribution of piRNA reads corresponding to sequences harbouring repeats and TEs. NRapiRNAs: Distribution of piRNA reads not associated with repeats nor TEs.

**Figure 3:**
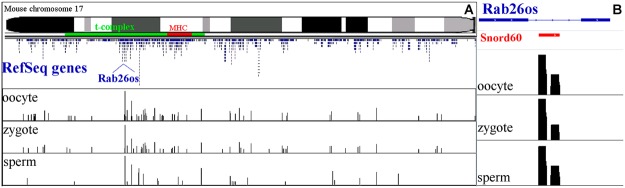
Mouse chromosome 17 NRapiRNA coverage. Panel A represents mouse chromosome 17 with the three cell type coverage tracks. Blue marks represent RefSeq annotated genes on chromosome 17. In green, the mouse *t*-complex region and in red the MHC gene region. Coverage tracks represented in black for each cell type. Panel B represents coverage tracks of the antisense lncRNA *Rab26os* including its intron-derived snoRNAs. Bars represent the log transformed coverage values obtained using the IGV software. Structural annotations of *Rab26os* were obtained from the Ensembl database.

We assessed the chromosome mapping distribution of the two piRNA populations with respect to both gamete types and zygotes (Fig. 2). The RapiRNA and NRapiRNA chromosome distributions in the mouse genome showed marked differences. Oocyte and zygote RapiRNAs presented a similar spread in its chromosomal distribution. However, a pattern with a higher representation of RapiRNAs on chromosomes 1, 8, 13, and X was detected in sperm cell (Fig. 2A). Conversely, NRapiRNA analysis showed by far that most of them mapped on chromosome 17 (Fig. 2B). What is more interesting is that the mouse *t*-complex is located on chromosome 17^38^, emphasizing that many major histocompatibility complex (MHC) genes are also present in this region. Coverage analysis of chromosome 17 evidenced that most of the NRapiRNAs mapped in this *t*-complex region (Fig. 3A). Most remarkable, the highest piRNA expression mapped on chromosome 17 to a precise intronic region of the *Rab26os* gene which also locates a small nucleolar RNA (snoRNA) (Fig. 3B), advocating a connection between snoRNA and piRNA^6^ as is described in the following section.

### Differential piRNA distribution in gene regions

**Figure 4:**
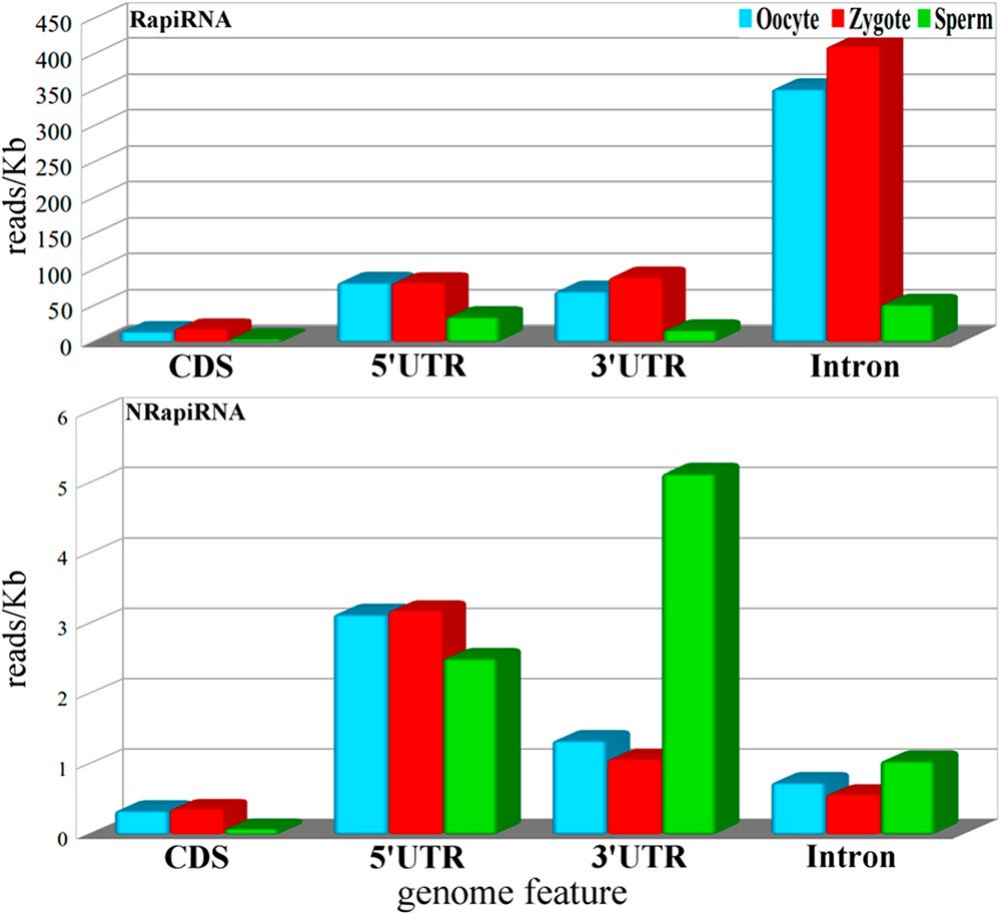
Genomic distribution of piRNAs associated and non-associated with repeat elements. Up-panel bar diagrams show the density distribution of read of piRNAs associated with repeat-element, whereas the down panel is displayed the piRNA non-associated with repeat elements (NRapiRNAs). piRNA density was calculated based on the number of the reads that mapped a feature according to Ensembl annotation divided by total bases present in these features.

To assess potential different functions based on the association of piRNAs to different functional gene regions, we analysed the piRNA mapping density (reads/kb) of different gene regions using RSeQC with the Ensembl annotation: coding regions, 5′ and 3′ untranslated regions (UTRs) and introns (Fig. 4). We separately analysed these distributions for the RapiRNA and NRapiRNA populations. Noteworthy, RapiRNA displayed differences in gene region distribution between samples (Fig. 4A). RapiRNA density was higher in intronic regions in oocytes and zygotes. On the other hand, NRapiRNA was detected to be more highly associated with 5′UTRs in all cell types, although sperm cells presented higher association at the 3′UTR (Fig. 4B). Again, this suggests functional differences between RapiRNA and NRapiRNA populations as well as male *versus* female gametes.

### Distribution of piRNA associated with intronic regions

**Figure 5:**
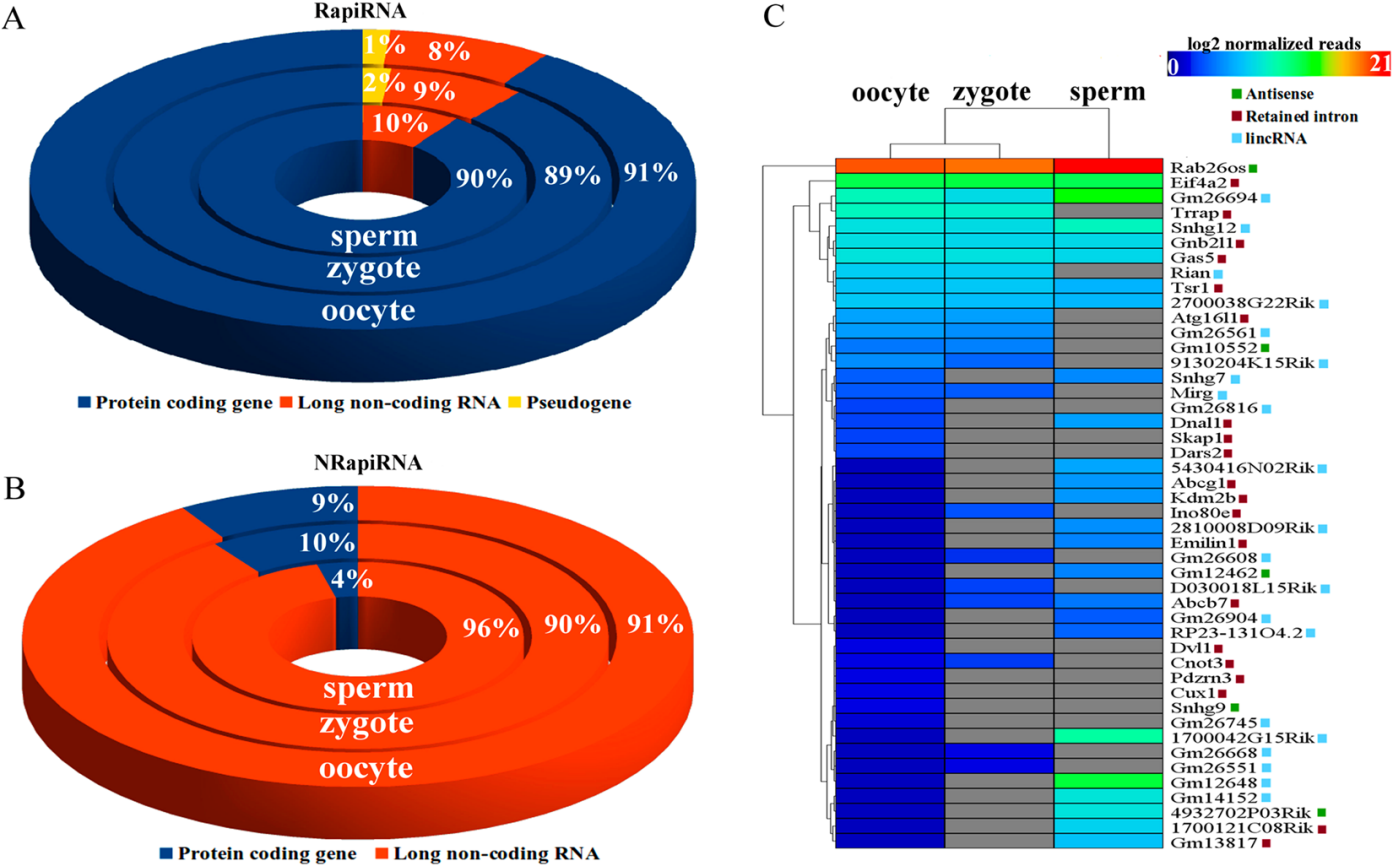
Annotation and classification of piRNAs mapping in introns. Circular diagrams show the piRNA read distribution associated with the intron of protein-coding genes, lncRNAs and pseudogenes. (**A**) RapiRNAs and (**B**) NRapiRNAs. (**C**) Hierarchical clustering of the intron mapping NRapiRNAs derived from lncRNAs. Annotation and lncRNA classification were based on the Ensembl database.

To delve into the potential functions of the piRNA derived from intronic regions of transcribed genes, we identify and classify transcripts using the Vega biotype classifications (http://vega.sanger.ac.uk). Independently of cell type, the vast majority of the intron-contained RapiRNAs mapped to introns of protein-coding genes (Fig. 5A). Moreover, RapiRNAs mapping to lncRNA or pseudogene introns showed a similar distribution pattern among the three cell types (Fig. 5A and Table S2). This also supports the idea that lncRNAs and pseudogenes or pseudogene-derived sequences could be a piRNA source^31^ due to TE presence in such introns^9^. Additionally, identification of piRNAs derived from antisense long non-coding RNA (alncRNA) in the three cell types hints to a potential participation of RapiRNA in connection with the gene regulatory functions of alncRNA^39^.

**Figure 6:**
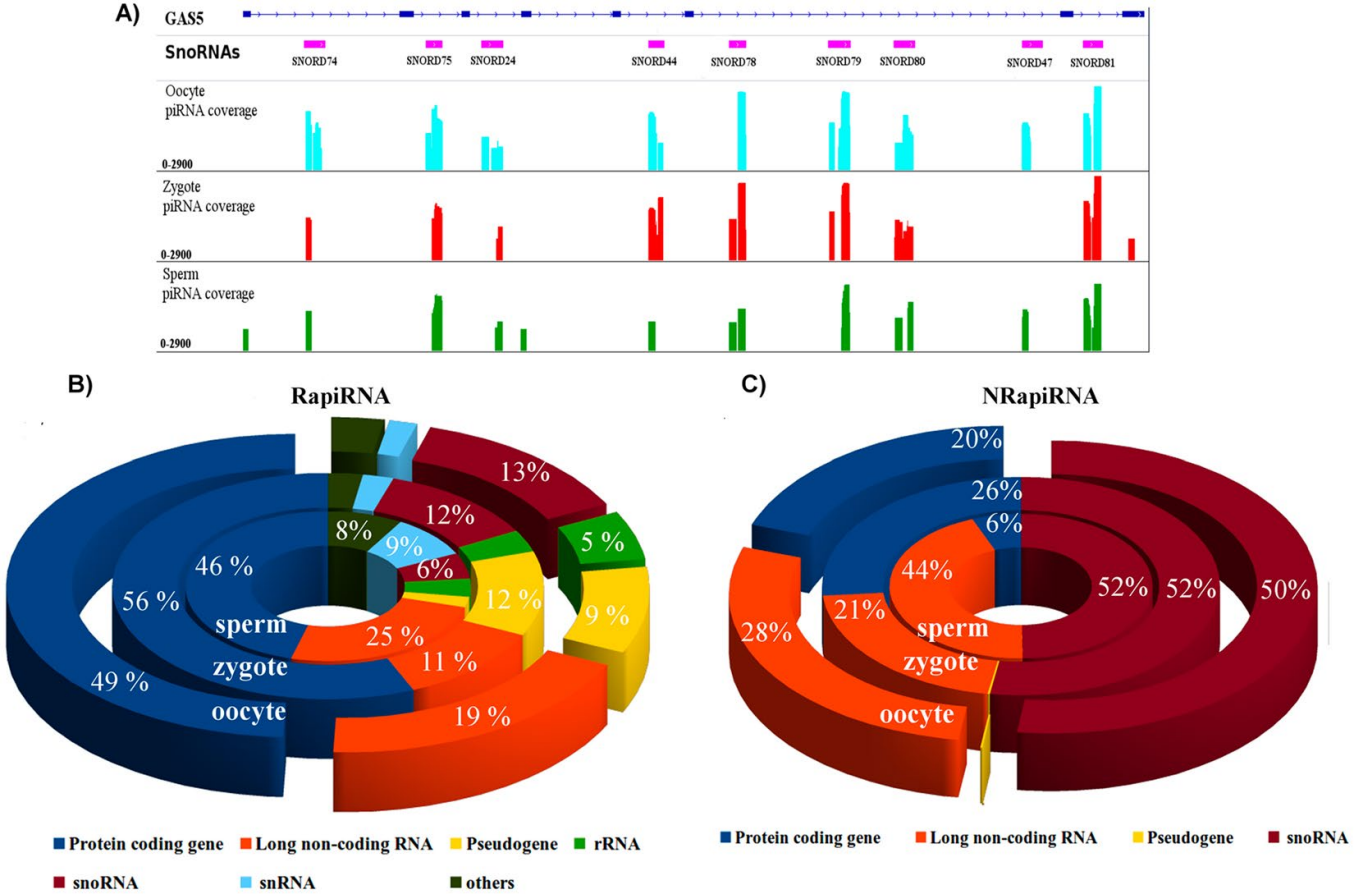
SnoRNA-derived piRNAs from lncRNA *GAS5* and piRNA associated with gene exons. (**A**) Coverage tracks of the identified piRNAs with respect to the three cell types. Bars represent the obtained log transformed coverage values using the IGV software. *GAS5* gene structure and snoRNAs were obtained from the Ensembl database. Circular diagrams show the exon associated RapiRNA read distribution (**B**) and NRapiRNAs (**C**). Biotype gene classifications according to the Ensembl annotation.

piRNAs derived from lncRNAs exhibited cell specific expression, suggesting potential functional differences. For example, as shown in Fig. 5, piRNA derived from intron of lncRNA *Rab26os* and *Gas5* were found highly expressed in the three cell types. Interestingly, *Rab26os* locates on chromosome 17 within the t-complex region (Fig. 3B) and *Rab26os* intron-derived piRNA were the high-expressed piRNAs in sperm cells. *GAS5* produced piRNA from the snoRNA presents in its introns in human cancer cells^25^. In this regard, we identified differential expression of piRNA derived from snoRNAs presents in *GAS5* introns (Fig. 6A) depending of the cell type. For example, zygotes did not express RapiRNA resulting from *snoRNA47*, but expressed RapiRNA arising from the last exon of *GAS5*. However, in oocytes an NRapiRNA derived from *snoRNA74* was specifically detected in *GAS5* (Fig. 6A). Differential expression between male *versus* female gametes detected in piRNAs derived from lncRNAs and snoRNAs, and even in relatively close cell types such as oocytes *versus* zygotes, suggests the existence of regulatory crosstalk between piRNAs, lncRNAs and snoRNAs.

### Distribution of piRNA associated with exonic regions

**Figure 7:**
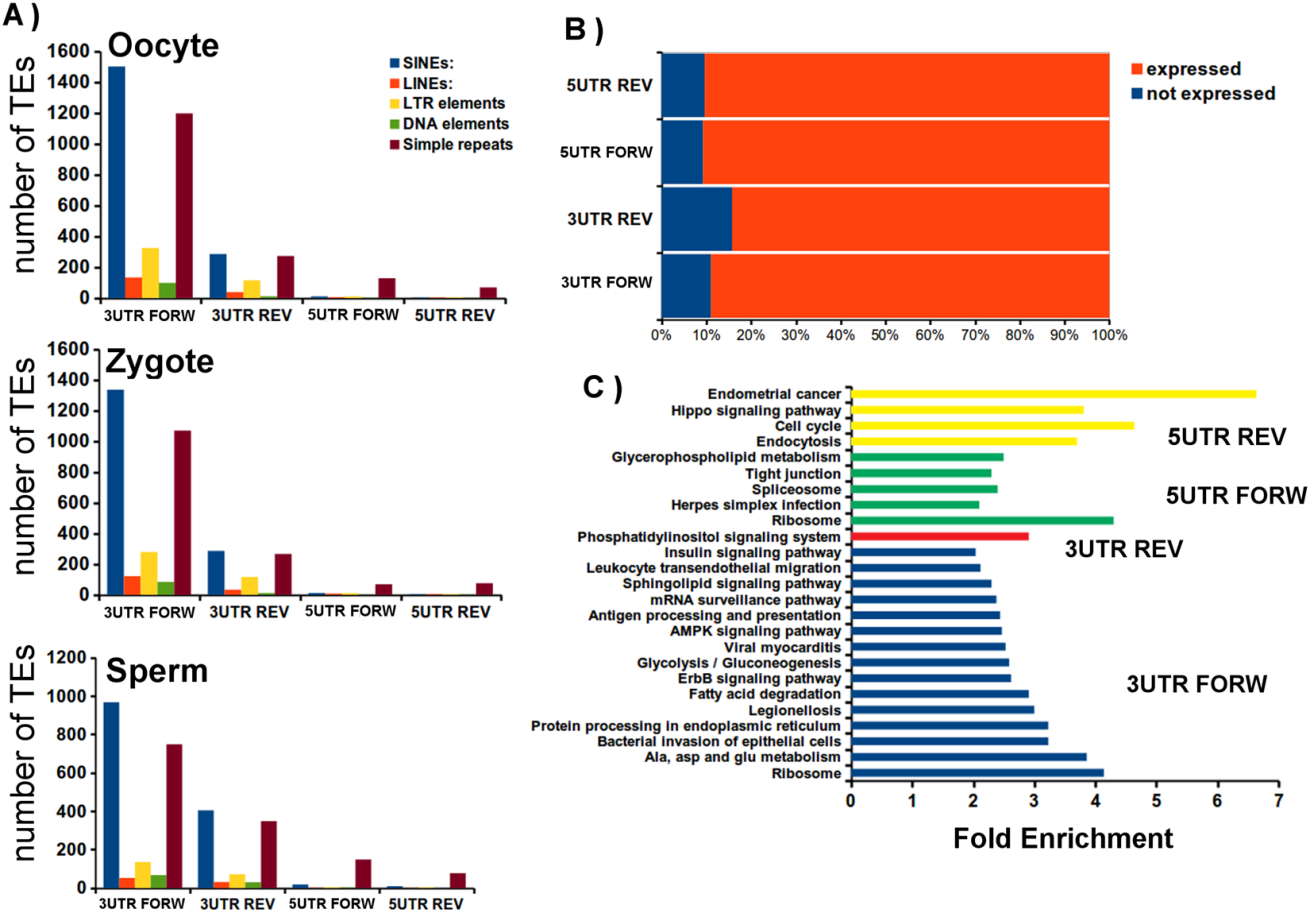
Analysis of the UTRs regions with associated RapiRNAs. Number of sequences of TEs and simple repeat found in UTRs sequences associated with RapiRNAs. (**B**) Graph represents the percentages of expressed/non-expressed genes and the UTRs regions with RapiRNAs-associated, (**C**) KEGG enriched analysis of genes expressed with RapiRNA-associated UTRs in spermatozoa.

To complete the analysis of association of piRNAs derived from gene regions, we characterized the piRNA population distributions across the exonic gene regions. In the RapiRNA population (Fig. 6B and Table S3) we found a wide diversity of piRNA derived from different transcript biotypes. Most remarkably, about half of the reads of the three cell types were associated with exons of protein-coding genes (Fig. 6B, and Table S3). Exons of lncRNAs represented the other abundant type of region associated to RapiRNA production. However, a reduction of RapiRNA expression from lncRNAs was detected in in the oocyte to zygote transition respect to piRNAs derived from protein-coding genes (Fig. 7A), which suggested some roles of these piRNAs in the cell progression after fertilization. Respective to the NRapiRNA population (Fig. 6C, and Table S3), we found that most piRNAs were associated with exons of snoRNA. These were underrepresented in the RapiRNA population (mainly in sperm cells). The NRapiRNA originating from coding genes reached to 20% and 26% for oocytes and zygotes respectively, but represented only 6% of sperm cell reads (Fig. 6A, and Table S3). Again, RapiRNA and NRapiRNA represented clearly differentiated association or biogenesis patterns with respect to both gametes and the zygote.

### piRNA population association with tRNA-derived RNA fragments

Recently, global associations between piRNA sequences with rRNA, snRNA, snoRNA and tRNA of gamete cells and zygotes have been described^6,24,26^. Our analysis has revealed that RapiRNA generated from tRNAs represented 54.4%, 20% and 13% of the total sperm, oocyte and zygote reads respectively. Our data confirmed previous analyses concerning the participation of tRNAs as tRFs (tRNA-derived RNA fragments) in piRNA generation^25,26^. Most noteworthy, the comparative supervised clustering analysis of the RapiRNA derived from expressed tRNA revealed a similar pattern in male and female germ cells (Fig. S3). The results confirmed that piRNA could be derived from other ncRNA molecules such as snoRNA or tRNAs^6,24^. Very recent studies suggest implication of tRFS in the control of TEs, being part of innate immune system of virus defence^40^.

### Analysis of piRNA-association to 3′ and 5′UTR regions of protein-coding genes

Presence of piRNA derived from the 3′UTR of mRNA has been previously detected in *Drosophila* ovaries, murine testes, and *Xenopus* eggs^29,30,33–43^ and also in mouse gonads and somatic cells^34^. We have identified piRNA mapping to the 5′ and 3′UTRs of transcripts, presenting fully complementarity sequence (Table 3 and Table S4). We approximately detected 400 and 200 mRNA sequences present as reverse oriented RapiRNA in the 3′ and 5′UTR respectively. In contrast, lower quantity of mRNA sequences was retrieved as antisense orientation in NRapiRNA with respect to both UTRs (Table 3). In addition, the analysis of the TEs families present in the UTRs with associated RApiRNAs is shown in the Fig. 7A. This analysis showed predominance of SINEs (short interspersed nuclear elements) in 3′UTRs regions associated to RapiRNAs in all the samples. (Fig. 7A). However, we have identified different protein coding genes with piRNAs associated with their 5′UTR regions (Table 3). The possible function of this piRNA associated to 5′UTRs for the moment it is unknown.

**Table 3:** Analysis of piRNAs associated with protein-coding 3′ and 5′UTR gene regions.

Number of protein-coding gene UTRs with	RapiRNAs			NRapiRNAs		
	oocyte	zygote	sperm	oocyte	zygote	sperm
5′ sense piRNAs	496	481	424	102	89	40
5′ antisense piRNAs	199	212	218	18	16	13
3′ sense piRNAs	1,216	1,199	724	395	366	98
3′ antisense piRNAs	425	448	374	27	18	13
**piRNA counts associated to potein-coding gene UTRs**	**oocyte**	**zygote**	**sperm**	**oocyte**	**zygote**	**sperm**
5′ sense piRNAs	87,232	88,238	53,248	14,186	13,116	31,033
5′ antisense piRNAs	19,483	14,835	12,296	464	401	1,019
3′ sense piRNAs	120,481	131,678	44,757	13,695	12,787	14,271
3′ antisense piRNAs	35,157	41,050	34,778	984	1,048	1,005

Only UTRs associated to more than 5 counts of normalized piRNA reads were considered.

Trying to identify a possible function of piRNAs in UTR regions, we performed a KEGG pathways enrichment analysis (Tables 4 and 5). Functional analysis of mRNAs harbouring RapiRNAs in 3′UTR in sense orientation revealed genes associated and related to the fertilization process, such as: estrogen signalling pathway and progesterone-mediated oocyte maturation appearing in oocytes and in zygotes (Fig. S5). However, enrichment analysis based on Mammalian Phenotype Ontology (MP) disclosed that the oocyte and zygote genes presenting RapiRNAs associated in 3′UTR sense orientation were related to abnormal embryological development and embryogenesis (Fig. S7A).

**Table 4:** Common enriched KEGG pathway transcripts with 3′ and 5′UTR associated RapiRNAs.

		3′UTR region						5′UTR region					
		sense			antisense			sense			antisense		
**KEGG Pathway**	**KEGG ID**	**oocyte**	**zygote**	**sperm**	**oocyte**	**zygote**	**sperm**	**oocyte**	**zygote**	**sperm**	**oocyte**	**zygote**	**sperm**
Adherens junction	mmu04520	2.6	—	—	—	—	—	3.9	—	—	—	—	—
Basal transcription factors	mmu03022	—	—	—	5.9	—	—	—	—	—	—	8.3	—
Cell cycle	mmu04110	—	1.9	—	—	—	—	—	—	—	4	—	4.3
Colorectal cancer	mmu05210	2.9	2.4	—	—	—	—	—	3.6	—	—	—	—
Endocytosis	mmu04144	—	—	1.7	—	—	—	—	2.1	2.3	—	—	3.5
Endometrial cancer	mmu05213	2.8	—	—	—	—	—	—	—	—	—	—	6.3
Epstein-Barr virus infection	mmu05169	1.5	1.7	—	—	—	—	2	—	—	—	—	—
Estrogen signaling pathway	mmu04915	2.5	2.5	—	—	—	—	3.3	2.9	—	—	—	—
Focal adhesion	mmu04510	1.8	—	1.7	—	—	—	—	—	—	3.1	3.5	—
FoxO signaling pathway	mmu04068	1.9	—	—	—	—	—	—	—	—	—	3.6	—
Glycerophospholipid metabolism	mmu00564	2.2	2.3	—	—	—	—	2.9	4.3	3.1	—	—	—
Herpes simplex infection	mmu05168	—	1.6	1.9	—	—	—	—	—	2.4	—	—	—
Lysosome	mmu04142	2.2	—	—	—	2.7	—	—	—	2.8	—	—	—
Oocyte meiosis	mmu04114	—	2.2	—	—	—	—	—	—	—	4.6	—	—
Pancreatic cancer	mmu05212	2.6	—	—	—	—	—	4.2	5.4	—	5.9	5.6	—
Pathways in cancer	mmu05200	1.7	—	—	—	—	—	1.8	1.9	—	2.9	2.7	—
Progesterone-mediated oocyte maturation	mmu04914	2.6	2.7	—	—	—	—	—	3.9	—	—	—	—
Regulation of actin cytoskeleton	mmu04810	2.—	1.6	—	—	2.5	—	—	—	2.1	—	—	—
Ribosome	mmu03010	2.7	3.2	4.3	—	—	—	4.9	4.8	4.7	—	—	—
RNA transport	mmu03013	2.7	3.—	—	3.1	3.5	—	2.2	2.7	2.3	—	—	—
Spliceosome	mmu03040	—	1.8	1.9	—	—	—	2.9	2.6	3	—	—	—
Tight junction	mmu04530	2.4	—	—	2.8	—	—	—	—	3.5	—	—	5.8

Numbers indicate the fold enrichment value of pathways after DAVID enrichment analysis using transcripts with RapiRNAs associated to their 3′ and 5′UTRs.

**Table 5:** Enriched KEGG pathway transcripts implicated in fertilization and immune system with 3′and 5′UTR associated NRapiRNAs.

		3′UTR region						5′UTR region		
		sense			Antisense			sense		
Common enriched KEGG Pathway	Pathway ID	oocyte	zygote	sperm	oocyte	zygote	sperm	oocyte	zygote	sperm
Homologous recombination	mmu03440	9.1	12.4	—	—	—	—	—	—	—
Signalling pathways regulating pluripotency of stem cells	mmu04550	4.6	3.0	—	—	—	—	—	—	—
Estrogen signalling pathway	mmu04915	4.6	3.5	—	—	—	—	—	—	—
Progesterone-mediated oocyte maturation	mmu04914	4.4	4.0	—	—	—	—	—	—	—
FoxO signalling pathway	mmu04068	4.3	3.1	—	—	—	—	—	—	—
T cell receptor signalling pathway	mmu04660	3.1	—	—	—	—	—	—	—	—
TNF signalling pathway	mmu04668	2.9	—	—	—	—	—	—	—	—
Platelet activation	mmu04611	2.9	—	—	—	—	—	—	—	—
Oxytocin signalling pathway	mmu04921	2.8	—	—	—	—	—	—	—	—
Thyroid hormone signalling pathway	mmu04919	2.8	—	—	—	—	—	—	—	—
Neurotrophin signalling pathway	mmu04722	—	3.4	—	—	—	—	—	—	—
Regulation of actin cytoskeleton	mmu04810	—	2.6	—	—	—	—	—	—	—
mRNA surveillance pathway	mmu03015	—	—	—	17.9	—	—	—	—	—
TGF-beta signalling pathway	mmu04350	—	—	—	—	—	—	10.4	9.8	—
Rap1 signalling pathway	mmu04015	—	—	—	—	—	—	5.2	—	—
Oocyte meiosis	mmu04114	—	—	—	—	—	—	6.0	—	—

Numbers indicate the fold enrichment value of pathways after DAVID enrichment analysis using transcripts with NRapiRNAs associated to their 3′ and 5′UTRs.

Enrichment analysis of mRNA targeted by NRapiRNAs in 3′UTR sense orientation suggests the participation of these mRNA in different cellular and developmental processes. In that regard, in oocytes and zygotes we also detected enrichment in pathways related to the oocyte maturation and fertilization such as meiotic processes, estrogen signalling pathway and progesterone-mediated oocyte maturation (Table 5). Interestingly, we detected enrichment pathways associated to the immune system process and mRNA stability in oocyte (Table 5).

Regarding RapiRNAs associated to mRNA in 5′UTR sense and antisense orientations, some common enriched pathways were identified in all samples, as well as specific pathways for male or female gametes, as for instance oocyte maturation (Table 4, Fig. S6). Analysis of mRNAs associated with RapiRNAs based on MP enrichment revealed their participation in embryo development (Fig. S7B,D). Particularly in the case of zygotes and sperm cells, MP analysis revealed enrichment genes associated to the immune system (Fig. S7B).

### Assessing of the association of RapiRNAs to UTRs regions of mRNA expressed in sperm

At the aim to validate our results, we have identified genes with RapiRNAs associated with UTRs expressed in sperm cells (Fig. 7B). More than 80% of the genes identified as RapiRNA associated with UTRs are expressed in mouse spermatozoa (Fig. 7B). The enrichment analysis of these genes (Fig. 7C) confirms the association of these genes with of the immune system, cell cycle and signaling pathways. However, the genes of phosphatidylinositol signalling are the only genes participating in the enriched pathways associated to piRNAs by complementarity in the 3′UTRs. Phosphatidylinositol phosphates (PIPs) play a crucial role in sperm development, and are crucial in the different stages of gamete differentiation. Altogether, these results supported potential piRNA roles as a post-transcriptional regulator of gene expression acting in the UTR regions of protein-coding mRNA.

## Discussion

In this study, taking forwards the bioinformatics analysis of previous NGS data, we have integrated and unmasked piRNA origins and pinpointed their specific gene and genomic region associations to discover new potential regulatory functions in germ cells and zygotes. For that goal, integrative piRNA immunoprecipitation databases were first updated to generate, to the best of our knowledge, a novel database containing the largest number of sequences obtained by PIWI protein immunoprecipitation^32^.

This new database, now an open source, has allowed us so far to identify a greater number of piRNA sequences than previously^6,24^, indicative that the piRNA repertoire is more extensive than previously uncovered in germ cells and zygotes. Our bioinformatics pipeline has enabled us to classify piRNA into two populations, namely: RapiRNAs and NRapiRNAs based on their genomic repeat and transposable element relationships. As expected, the repeat associated RapiRNAs were basically coupled to TE regions. However, the data obtained suggest that some piRNAs could participate in other biological functions, such as in the regulation of protein-coding genes via piRNA interactions with their 3′ or 5′UTRs, similar to the previously reported results obtained in marmoset testis^41^ and human somatic and cancer-type tissues^41^. In addition, functional interactions between piRNAs and TEs could also participated in oocyte gene regulation^42^.

By contrast, this suggested that NRapiRNAs, non-associated to TEs or any other genomic repeat motifs, might be involved in different biological processes completely independent of TE genome defence (initially reported as the major function of piRNAs). The NRapiRNA proportion in gametes and zygotes was relatively low and similar to that detected in somatic tissues^19,34^. Consequently, in germ cells and the zygote piRNAs could contribute to differentiation of gameta and early development with dual potential roles: as canonical defense of transposable of TEs in the germline as well as epigenetic and/or post-transcriptional regulation of define genes.

piRNA populations identified using our bioinformatics pipeline displayed sequence length distribution differences, sequence bias in the first 5′ nucleotide and chromosome mapping dissimilarities.

Regarding the piRNA chromosomal distributions, noteworthy the NRapiRNA population association with mouse chromosome 17. We deem particularly important that this proximal region of chromosome 17 also contains the major histocompatibility complex (MHC) (Fig. 3) composed of 11 antigen subclasses, comprising in mouse and human genomes 88 genes encoding molecules involved in the initiation of the immune system response^43^. Interestingly, Kawano and collaborators^44^ demonstrated the presence of methylated sperm RNAs (spR), associated to piRNA cluster in chromosome 17, implicated in early embryogenesis^44^. Major histocompatibility complex (MHC) (HLA in humans) plays key roles in the immune response. In mammals, both sperm and early embryos lack elements of the MHC. Hence, the uterus tolerates the allogeneic sperm cells and the semiallogeneic zygote and pre-implantation embryo. How MHC genes are downregulated in these cells is still unknown. In this sense, the surprising detection of piRNAs mapping to 33 genes of the MHC is particularly significant considering the potential implications that the interaction of these specific piRNAs with MHC genes may have facilitating an explanation to the Medawar paradox brought in 1953^45^. The identification in sperm cells of additional piRNAs associated with genes participating in pathways involved in the immune system processing, such as antigen processing and presentation (Fig. 7C), reinforces this hypothesis that will require further studies.

The genomic distributions of piRNAs from the two types of piRNA populations presented differential genomic associations. RapiRNAs associated preferentially to introns of different lincRNAs, as well as to introns of protein-coding genes were uncovered. Noteworthy, TEs were preferentially fixed in a fast-evolving manner to certain lincRNA genes of mammalian genomes^9^. These results are consistent with the idea that TEs are not randomly distributed throughout of the genome, but have suffered a selective pressure to undergo an uneven genomic distribution with a concomitant co-evolution of piRNAs and TEs^46^. The insertion of TEs in introns of protein-coding genes has been identified to be involved in the generation of new regulatory functions. These relationships between gene and transposable element have also been characterized in human testes^30,31^. Results have suggested the possibility that piRNAs could comprise mediating molecules regulating methylation of specific loci. This hypothesis is based on the ability of the PIWI-piRNA complex to interact with the methylation-demethylation machinery. Indeed, different PIWI proteins have their own methylation targets^47^. Nevertheless, the NRapiRNA population associations to introns were more frequent in relation to alncRNAs. In particular, cell types revealing a strong NRapiRNA association with the alncRNA *Rab26os* proved to contain *snoRNA60* in its intron. *Snord60* is expressed equally high in human ovaries and testes (over a 10 fold of the expression in heart), as well as in pooled germ cell tumours^48^. Along with *Snord60* participation in cholesterol intracellular traffic^49^, other functions of this snoRNA remain unknown.

Our genomic analysis related to exon-associated piRNAs has confirmed their association with snoRNAs and rRNAs, together with snRNAs, which we had previously described as present in male germ cells^6^. Indeed, we detected piRNAs associated to snoRNAs and present in lincRNA *GAS5*. In human cancer cells, the piRNA overexpression derived from snoRNA in *GAS5* induces transcriptomic changes at the promoter level^25^. The long non-coding *GAS5* gene contains 9 introns transcribing snoRNAs in mouse, being these snoRNAs the only conserved regions between the mouse and the human *GAS5* homolog, thus suggesting important functions for these snoRNA-derived piRNA molecules. Among them, recent studies carried out in human CD4 T-lymphocytes showed that piRNAs produced from snoRNAs can regulate gene expression via sequence complementarity with an interleukin-4 (*IL-4*) intron, leading to the decay of targeted pre-mRNA through nuclear exosomes^50^. These findings open the door to new putative functions of these snoRNA-derived piRNA molecules as to gene expression regulation.

Previous studies have evidenced that the piRNAs can regulate protein-coding genes at the 3′UTR^15,29,33^. For this reason, we analysed the association of piRNA classified into the RapiRNA and NRapiRNA populations with the UTR regions of protein-coding genes. piRNA association analysis to 3′UTR regions of protein-coding genes expressed in gametes and zygotes indicated existence of an important 3′UTR associated piRNA bias. However, some piRNAs associated with 5′UTR were also identified in a large proportion of the cases functionally associated to the gametogenesis and fertilization processes (Tables 4 and 5) by gene ontology. piRNA associations with 5′UTR regions remain not well understood, yet has been suggested as participating in gene expression regulation. Notably, it has been reported that the memory regulation in *Aplysia* is controlled by the interaction of piRNAs with the 5′UTR region of the *CREB2* gene^51^.

Finally, we explored in gametes and zygotes the piRNA capacity to interact in antisense with protein-coding genes in the same manner that TE expression is regulated. The participation of piRNAs to silence different expressed genes from the spermatogenesis process is known^52^. We found piRNAs mapping to the 3′UTR of several genes might control different pathways associated to the fertilization process by silencing mRNAs in a similar way as miRNAs accomplish, but with different dynamic considering the lack direct correlation between piRNA expression and the corresponding potential targets as has been reported in *Drosophila* and mouse^29^. In addition, we have identified that some piRNAs associated to the 3′ and 5′UTR regions are involved in the same pathways, thus suggesting the possibility that some piRNAs could participate in the regulation of similar processes by different ways.

Our integrative analysis also focused on the genomic origins of some piRNA molecules, suggesting new piRNA biogenesis and roles in gametes and zygotes, beyond the purpose of transposable sequence repression. Hence, the regulation of transposable elements by piRNAs in genome defence appears not to be their exclusive role. The origin of piRNAs derived from snoRNAs and tRNAs has also been confirmed, paving new potential piRNA functions, such as the regulation of gene expression at different transcriptomic levels.

## Methods

### NGS data collection

Small RNA-seq libraries derived from oocytes, sperm cells and zygotes were obtained from previous our NGS data deposited in the GenBank RSA archive (accession numbers SRX648519-21). Generation of these small RNA-seq libraries and trimming of adapters have been described in^6^. The trimmed reads were collapsed and sequences with five occurrences or less were removed (cleaning process). We initially obtained NGS data of sncRNAs from CD-1 mice. All animals were used in strict accordance with the recommendations laid out by the Spanish Royal Legislative Decree RD53/2013 for the Care and Use of Laboratory Animals. The Animal Experiments Bioethics Committee of the Centro de Investigaciones Biológicas (CSIC) approved the protocol (Permit: CAM/PROEX 054/15). All procedures for handling animals were performed in accordance with the regulations of the European Commission (Directive 2010/63/UE and Directive 86/609/ECC) and all efforts were made to minimize their suffering while reducing the number of animals.

### Construction of a collective piRNA database from immunoprecipitation data

Following the definition of piRNAs as those sncRNAs interacting with PIWI proteins, we considered as piRNAs the small RNAs that were identified as bound to PIWI and consequently recovered by immunoprecipitation approaches^13,14,29^. We used this criterion to create a new integrated database derived from the piRBASE^32^. We downloaded the piRNA sequences from datasets deposited in the piRBASE generated by immunoprecipitation of the different mouse PIWI proteins. The piRNA sequences downloaded from the datasets were collapsed and filtered in order to avoid duplicate sequences. The different datasets used to generate this specific immunoprecipitation piRNA database (IPpiRNA db) are resumed in Table S1. As a result, the IPpiRNA db contained 16,033,825 unique piRNA sequences. piRNA lengths and nucleotide distributions of the database are displayed in Fig. S1. The IPpiRNA-db database is available at: https://github.com/edugenetico/Immunoprecipitation-piRNA-database.

### Identification of piRNAs from the small RNA-seq libraries

The bioinformatics pipeline analysis of the small RNA-seq libraries is described in Fig. S2. All of the mapping steps indicated in the bioinformatics pipeline were performed using the short read aligner Bowtie 1.1.2. (http://bowtie-bio.sourceforge.net/index.shtml) considering parameters *tryhard*, *strata*, *best* and *chunkmbs 256*. Clean reads were mapped against the miRNA precursor (pre-miR) sequences downloaded from the mirBase version 21 (http://www.mirbase.org/ftp.shtml) and miRNA isoforms (termed as “isomiR”) obtained from Isomirage^53^ applying Bowtie while allowing for 3 mismatches (v 3). To identify piRNAs, reads not mapped to the different miRNA databases were used to map against the IPpiRNA database making use of a Bowtie 0 mismatch allowance (v 0) and a first best alignment (k 1) report. Reads identified as piRNA were mapped to the mm10/GRCm38 mouse genome assembly allowing for 3 mismatches (v 3) whilst reporting the one hundred best alignments (k 100). Due to nucleotide variant differences found among the genome sequences of different mouse strains^54^ and owing to the outbred nature of the CD1 mouse strain, we performed the mapping to the mouse genome allowing for 3 mismatches. A multi-mapping correction was made dividing the sequence read count by the number of times it mapped in the genome.

### Databases, genomic information and additional software used for the bioinformatics pipeline

The genomic feature distribution was calculated using the RSeQC package^53^. The mouse genomic sequences and the gene models were obtained from the Ensembl and UCSC genome browser databases (GRCm38.78/mm10), respectively. The mouse genomic sequences of the TEs and repeats (rmskJoinedBaseline track) were downloaded from the UCSC Genome Browser using the UCSC Table Browser. The genomic regions of introns as well as the 3′UTR and 5′UTR were downloaded from the UCSC Table Browser using the Ensembl gene annotation. Transcripts annotated in the UCSC of the same gene with different non-coding regions were considered as a single gene by collapsing the corresponding introns, 3′UTR or 5′UTR, making use of the intersect function of BedTools. Read counts of each genomic interval were obtained using the string method from HTSeq package^55^. Normalization of the raw counts was carried out with DESeq tool package^56^, using size factors values from each sample dataset. Only features with more than 5 normalized read counts were considered for the analysis. Interspersed repeats and TE families were identified using RepeatMasker (http://www.repeatmasker.org/). Sperm mRNA expression was downloaded from Mammalian Transcriptomic Database (MTD, http://mtd.cbi.ac.cn/index.php). Gene annotations were retrieved from the Mouse Genome Informatics (MGI, http://www.informatics.jax.org/). Visualization of the genomic datasets was performed using the Integrative Genomics Viewer (IGV). The enrichment analysis of the KEGG pathways was carried out using the DAVID Functional^57,58^ and enrichment (http://amp.pharm.mssm.edu/Enrichr/) tools^59^.

## Electronic supplementary material

Supplementary Information

## Data Availability

All data generated or analysed during this study are included in this published article [and its supplementary information files were downloaded from GitHub repository].
