# Supplementary material for: An integrative piRNA analysis of mouse gametes and zygotes reveals new potential origins and gene regulatory roles
Source: Sci Rep. 2018 Aug 27;8:12832. doi: 10.1038/s41598-018-31032-1 (PMC6110870; doi:10.1038/s41598-018-31032-1)
Supplement: Supplementary file 1 — Supplementary Information [file 41598_2018_31032_MOESM1_ESM.pdf]

An integrative piRNA analysis of mouse gametes and zygotes reveals new potential origins and gene regulatory roles.

Eduardo Larriba & Jesús del Mazo

Supplementary information

Supplementary Figure 1. Sequence lengths and nucleotide distributions of the immunoprecipitation piRNA database

A)

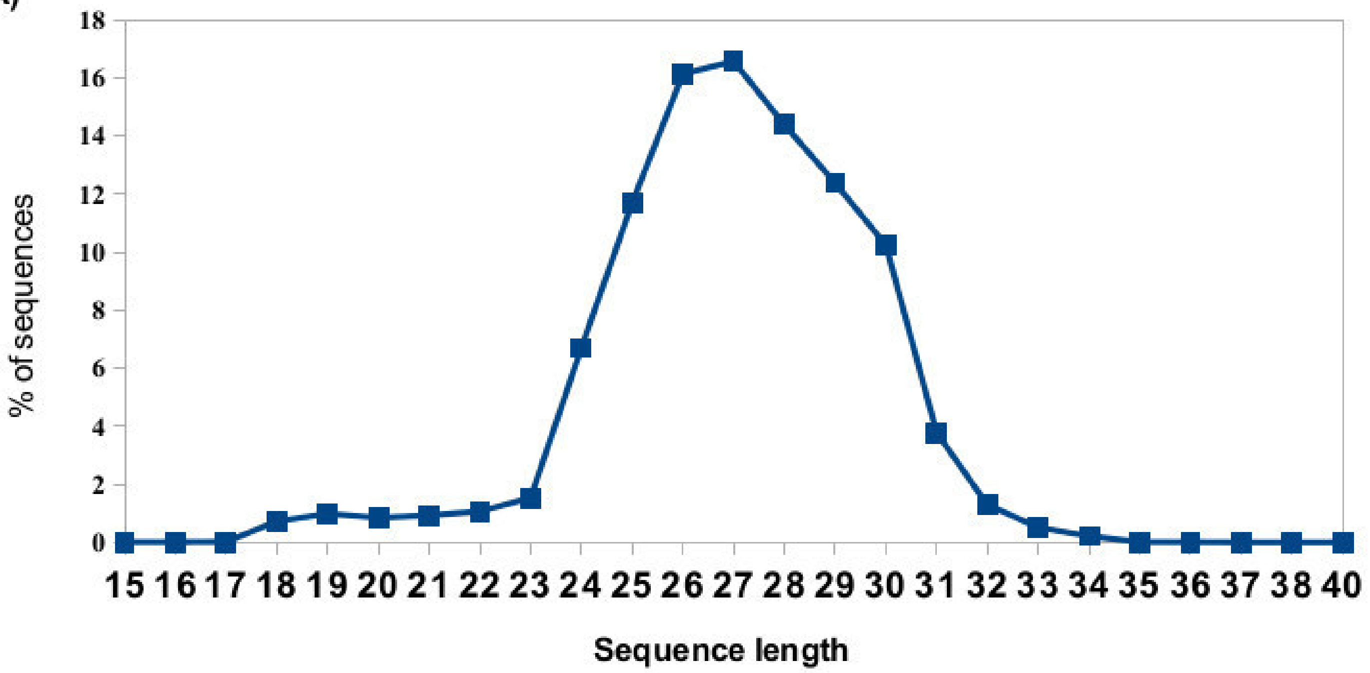

B)

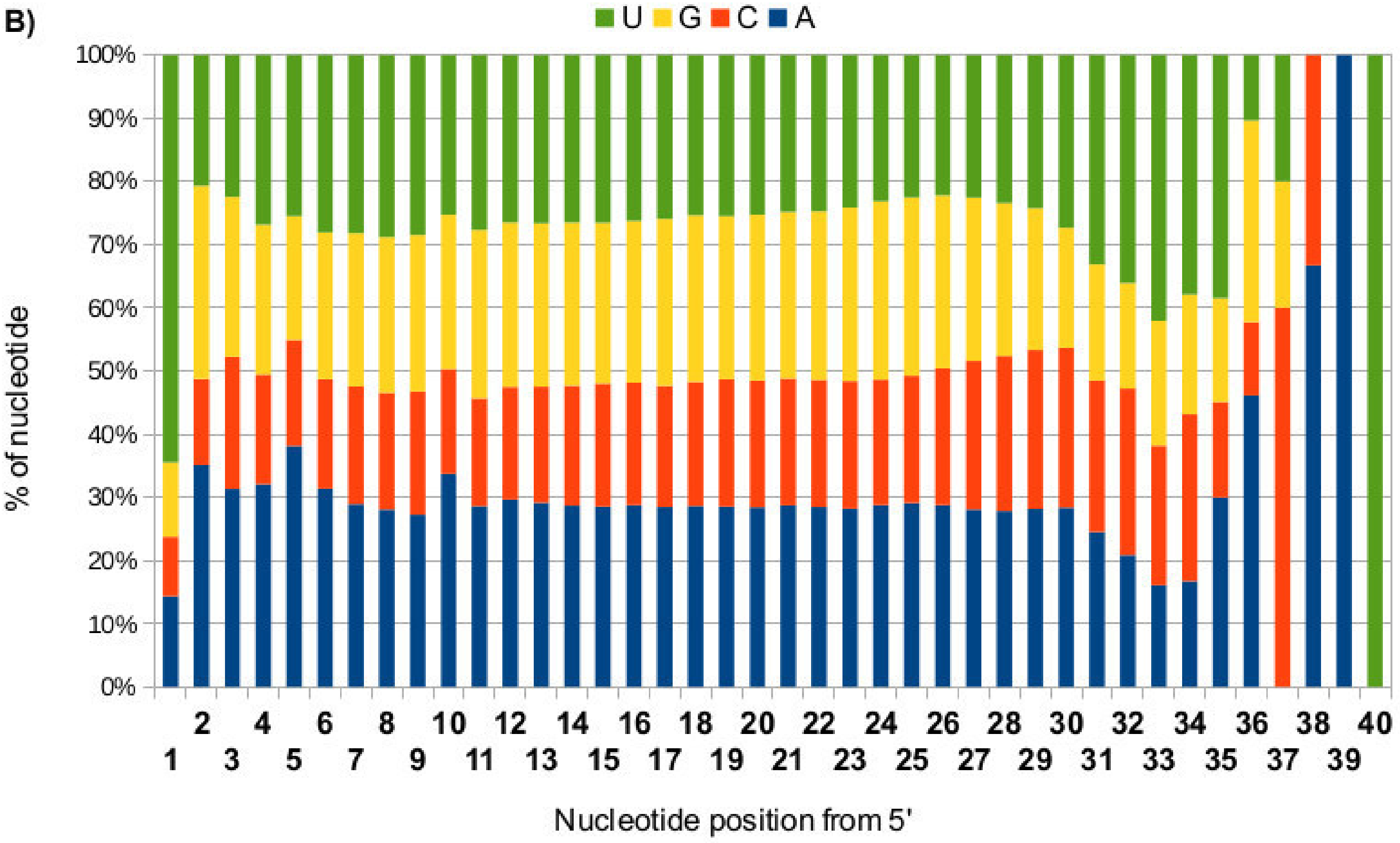

A) Graph shows the classified sequence percentages according to length. Sequence lengths consistent with the IPpiRNA database were calculated using the basic_analysis.pl script of the small RNA group package (http://www.anthropologie.uni-mainz.de/487_ENG_HTML.php). B) Nucleotide frequencies of the piRNAs sequences contained in the IPpiRNA database. Sequence percentages shown in relation to the total number of sequences appearing in the database (16,033,825 sequences).

Supplementary Figure 2. Small RNA-Seq library piRNA bioinformatics pipeline analysis

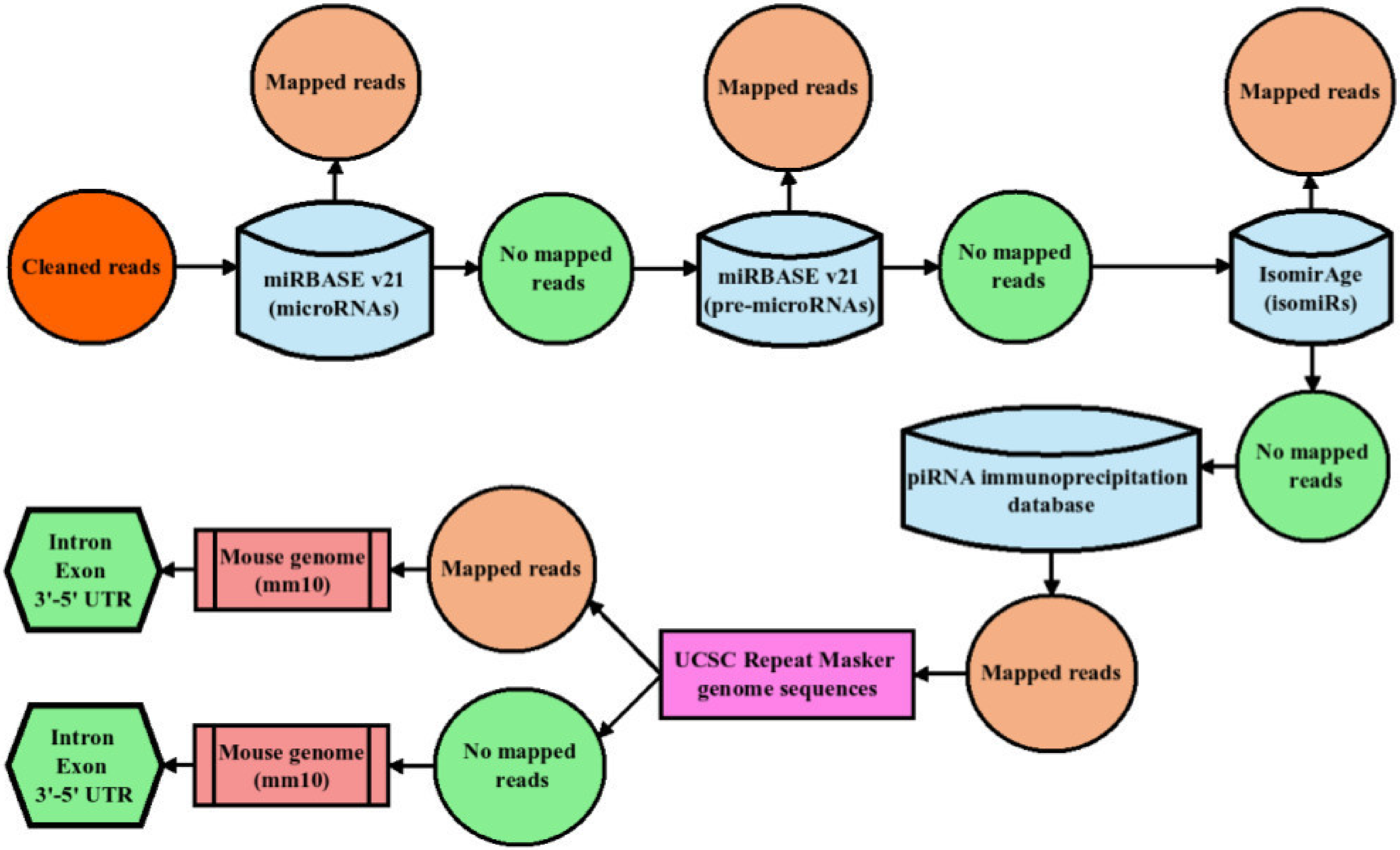

Bioinformatics workflow to identify piRNAs, classification and genomic structural annotation of sncRNAs.

Supplementary Figure 3. tRNA derived piRNA hierarchical clustering regarding oocytes, zygotes and sperm cells.

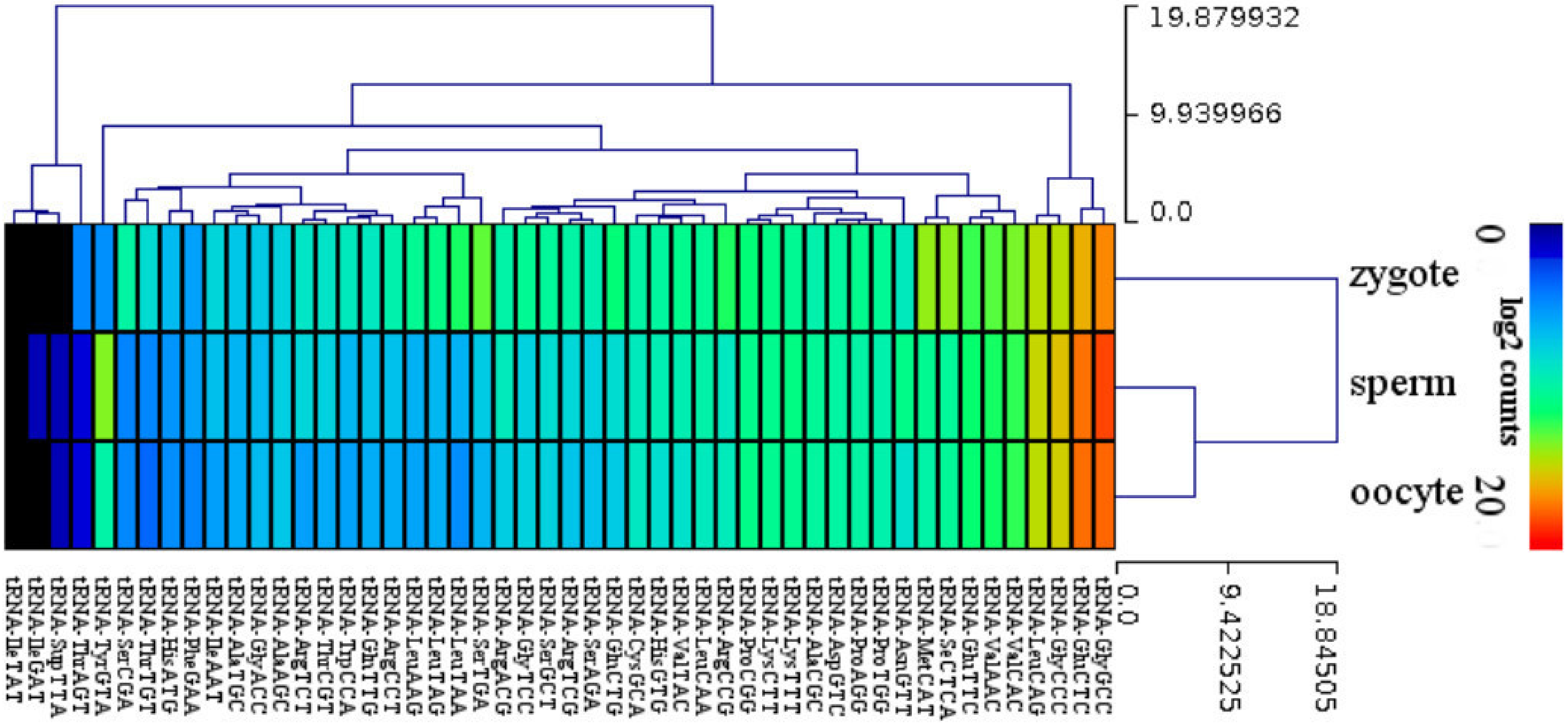

Supplementary Figure 4. Venny diagram of UTRs regions containing piRNAs with reference to oocytes, zygotes and sperm cells

A) RapiRNA

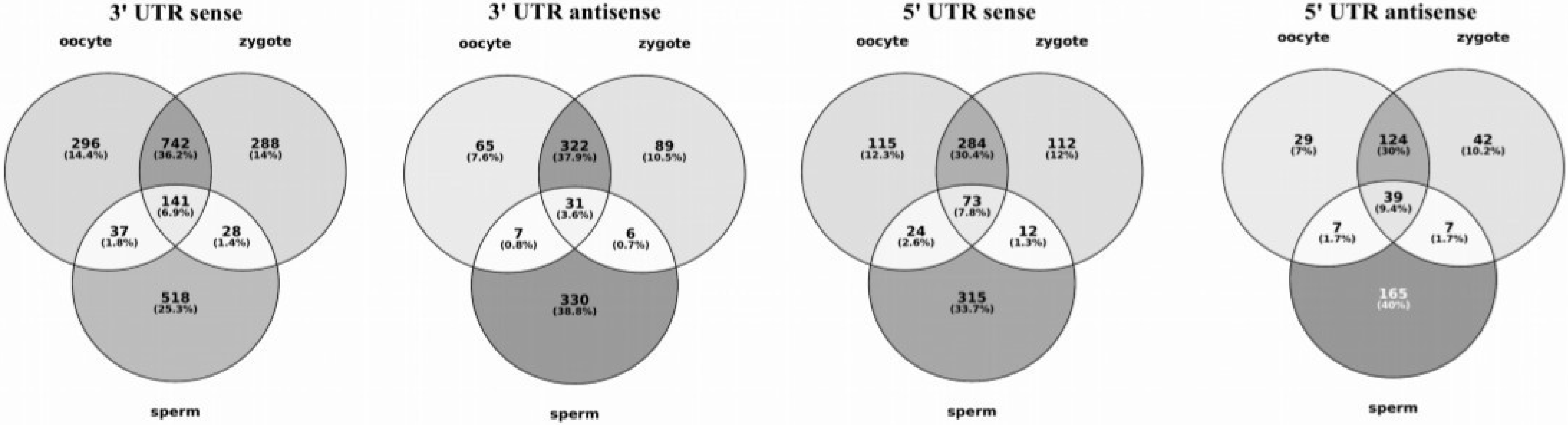

B) NRapiRNA

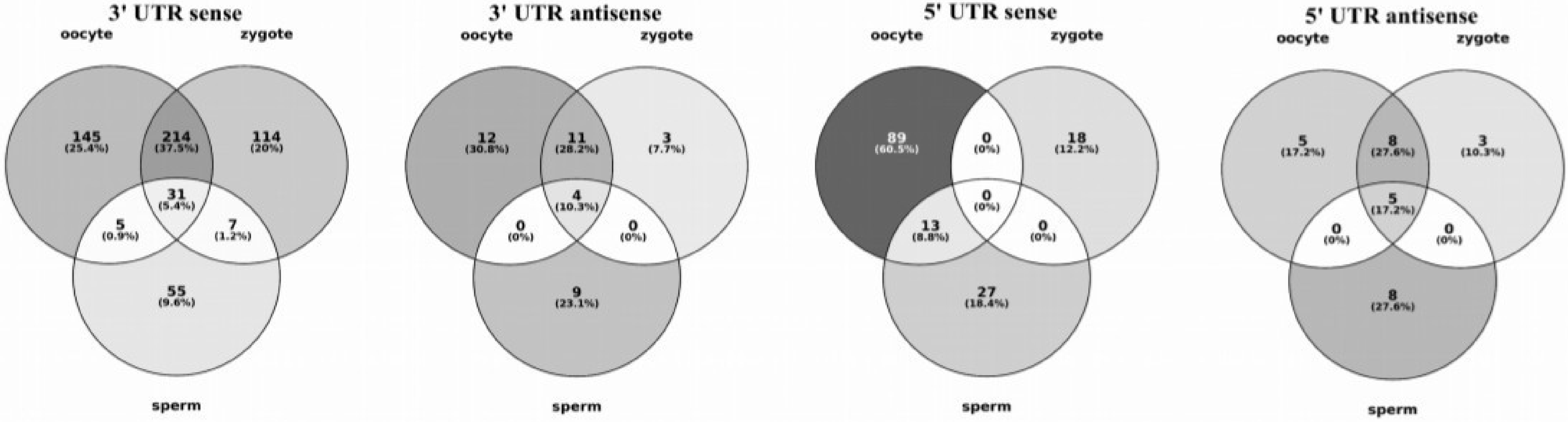

Supplementary Figure 5. KEGG enrichment analysis of transcripts including RapiRNAs in their 3'UTR regions

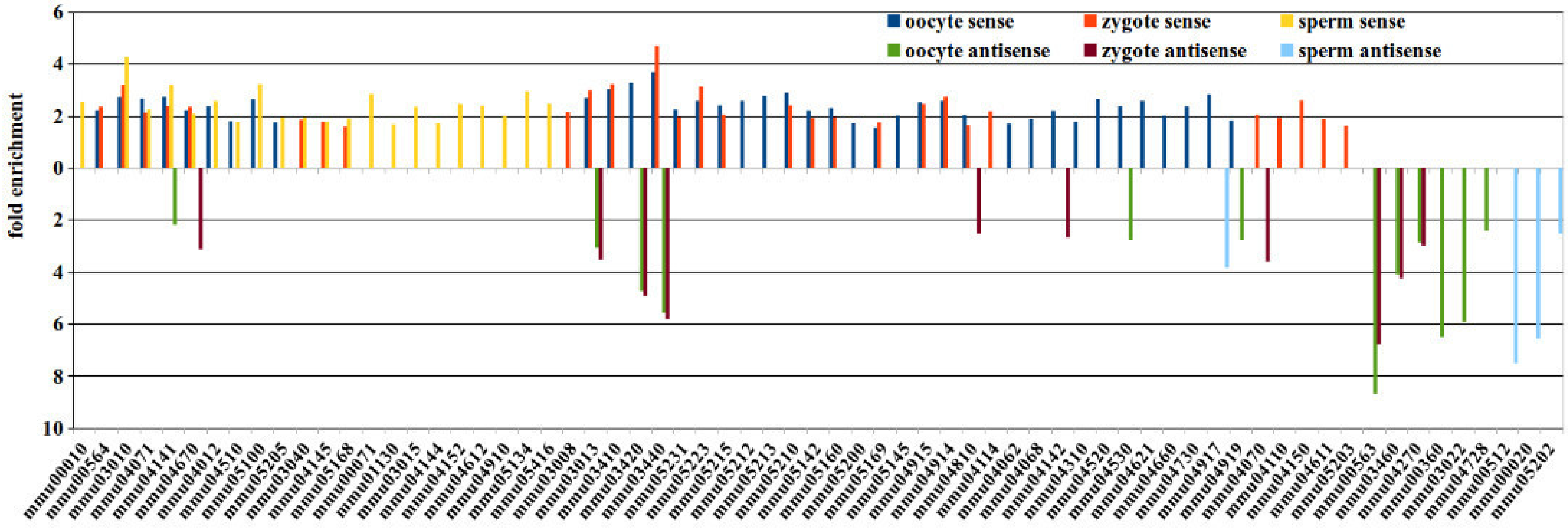

Supplementary Figure 6. KEGG enrichment analysis of transcripts containing RapiRNAs in their 5'UTR regions

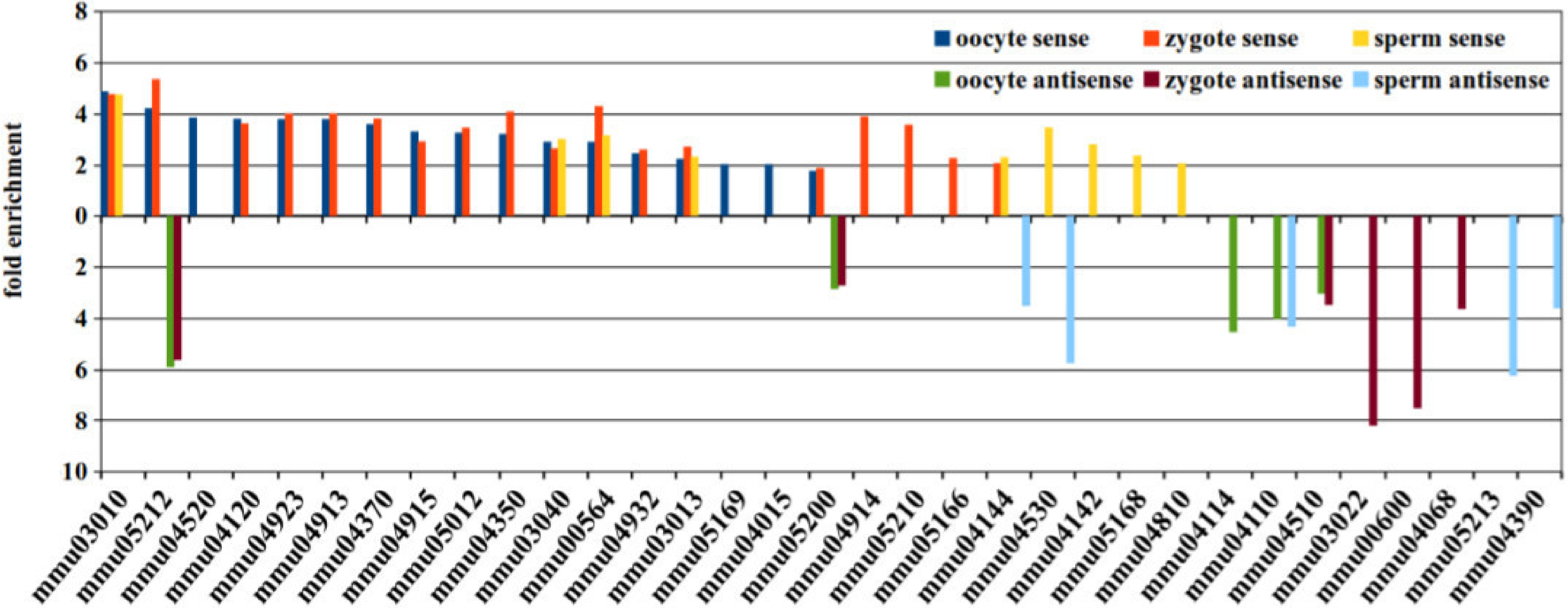

Caption:

KEGG ID	Pathway	KEGG ID	Pathway
mmu00564	Glycerophospholipid metabolism	mmu04520	Adherens junction
mmu00600	Sphingolipid metabolism	mmu04530	Tight junction
mmu03010	Ribosome	mmu04810	Regulation of actin cytoskeleton
mmu03013	RNA transport	mmu04913	Ovarian steroidogenesis
mmu03022	Basal transcription factors	mmu04914	Progesterone-mediated oocyte maturation
mmu03040	Spliceosome	mmu04915	Estrogen signaling pathway
mmu04015	Rap1 signaling pathway	mmu04923	Regulation of lipolysis in adipocytes
mmu04068	FoxO signaling pathway	mmu04932	Non-alcoholic fatty liver disease (NAFLD)
mmu04110	Cell cycle	mmu05012	Parkinson's disease
mmu04114	Oocyte meiosis	mmu05166	HTLV-I infection
mmu04120	Ubiquitin mediated proteolysis	mmu05168	Herpes simplex infection
mmu04142	Lysosome	mmu05169	Epstein-Barr virus infection
mmu04144	Endocytosis	mmu05200	Pathways in cancer
mmu04350	TGF-beta signaling pathway	mmu05210	Colorectal cancer
mmu04370	VEGF signaling pathway	mmu05212	Pancreatic cancer
mmu04390	Hippo signaling pathway	mmu05213	Endometrial cancer
mmu04510	Focal adhesion		

Supplementary Figure 7. Enrichment analysis of potentially target genes by RapiRNAs in their UTR regions with respect to MGI phenotypes

A) 3' UTR in sense

oocyte

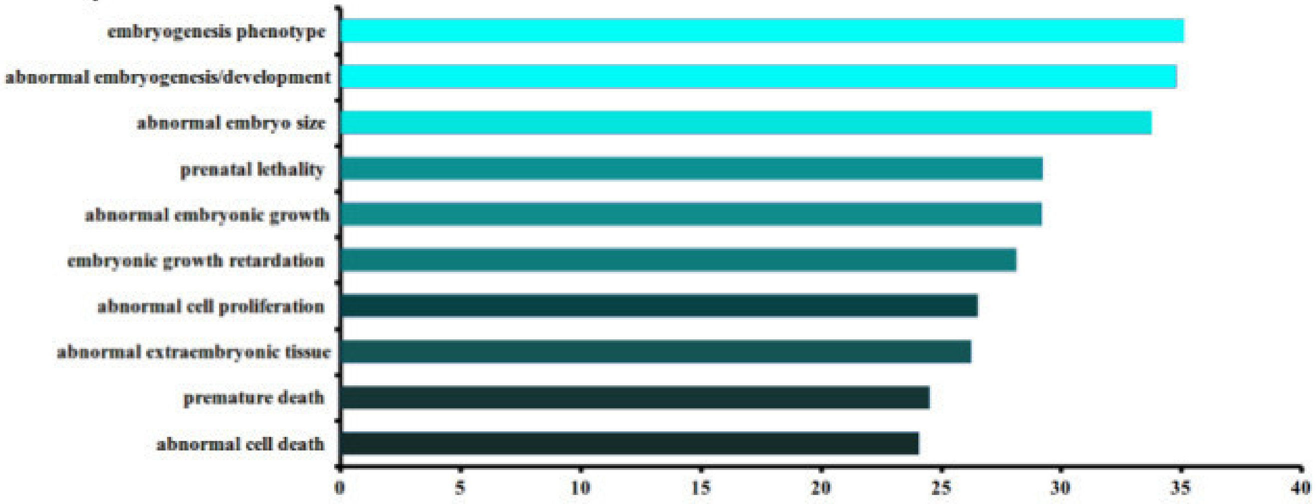

zygote

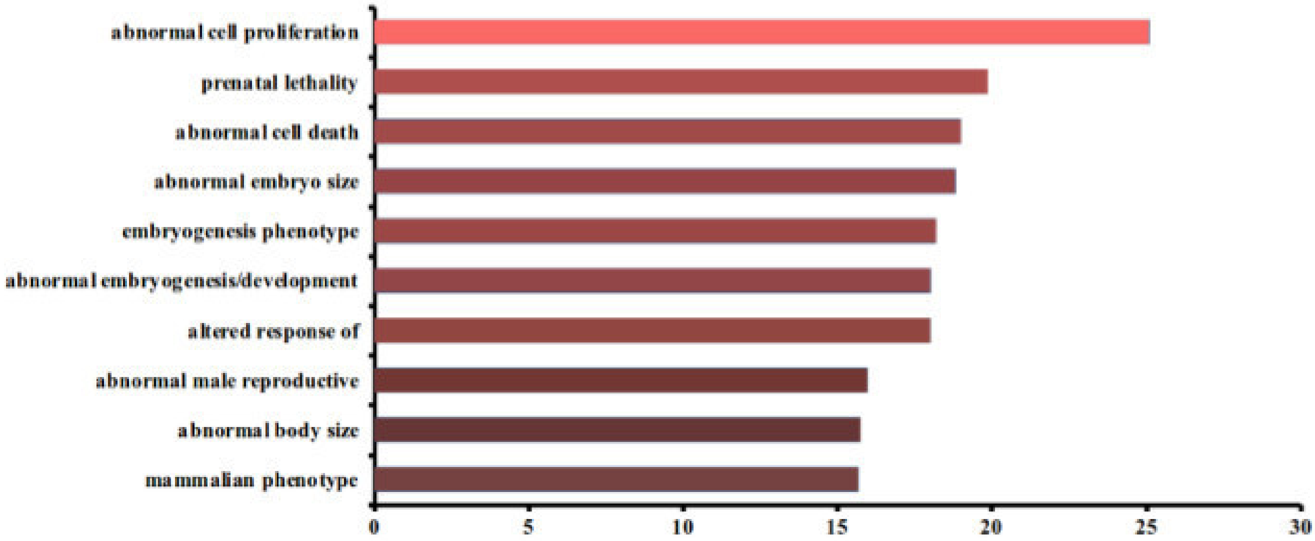

sperm

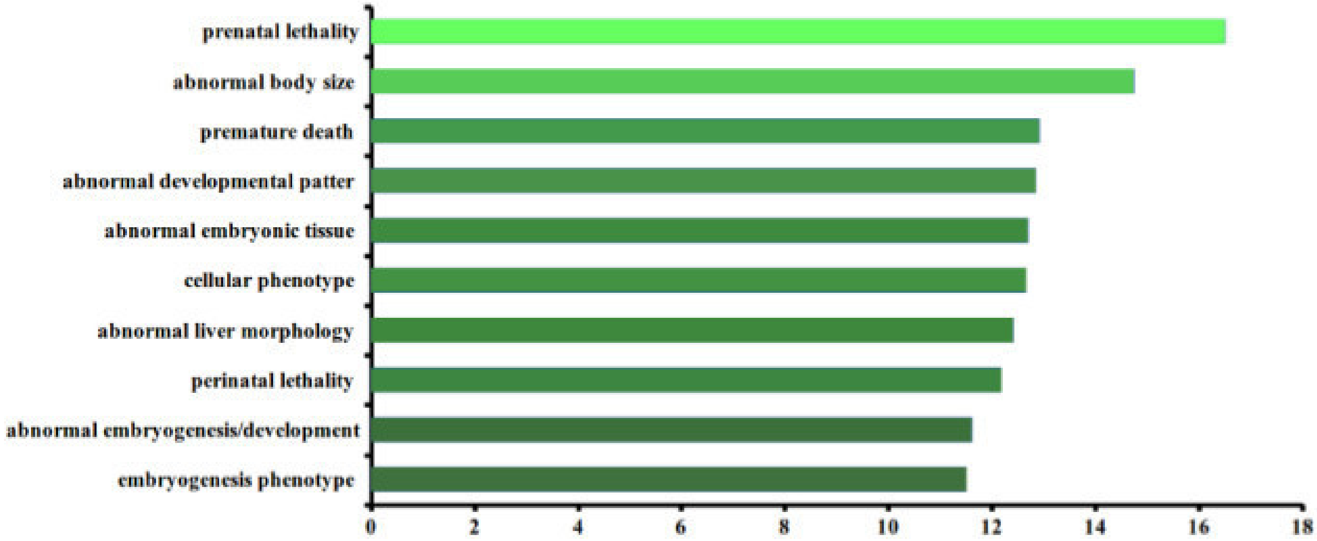

Supplemental Table 1. Datasets of the piRBASE databases used to generate the IPpiRNA db used in this study.

piRBASE ID	PubMed	Accession	Series	Uniq Seq	Method	Tissue
5	16751777	N/A	N/A	3482	MILI IP	testis
11	22121019	GSM822760	GSE32184	568759	Miwi IP	Testes, C57BL/6 Adult Miwi +/+
12	22121019	GSM822758	GSE32184	720903	Miwi IP	Testes, C57BL/6 P14 Miwi +/+
13	22121019	GSM822759	GSE32184	575786	Miwi IP	Testes, C57BL/6 P20 Miwi +/+
31	22842725	GSM684624	GSE27622	1068849	Miwi CLIP	C57BL/6 adult testis
32	22842725	GSM684625	GSE27622	464287	Miwi CLIP	C57BL/6 adult testis
33	22842725	GSM684626	GSE27622	118015	Miwi CLIP	C57BL/6 adult testis
34	22842725	GSM684627	GSE27622	129214	Miwi CLIP	C57BL/6 adult testis
35	22842725	GSM684620	GSE27622	249590	Mili CLIP	C57BL/6 adult testis
36	22842725	GSM684621	GSE27622	187442	Mili CLIP	C57BL/6 adult testis
37	22842725	GSM684622	GSE27622	167997	Mili CLIP	C57BL/6 adult testis
38	22842725	GSM684623	GSE27622	194293	Mili CLIP	C57BL/6 adult testis
60	18922463	GSM319956	GSE12757	334652	Mili IP	16.5 dpc testis
61	18922463	GSM319957	GSE12757	1086617	Miwi2 IP	16.5 dpc testis
72	17446352	GSM179088	GSE7414	136417	Mili IP	10 dpp testis
81	22020280	N/A	E-MTAB-730	2880834	Mili IP	wild_type_1 E16.5 fetal testis
82	22020280	N/A	E-MTAB-730	3229065	Mili IP	wild_type_2 E16.5 fetal testis
87	22020280	N/A	E-MTAB-730	1883101	Miwi2 IP	wild_type_1 E16.5 fetal testis
88	22020280	N/A	E-MTAB-730	1795983	Miwi2 IP	wild_type_2 E16.5 fetal testis
121	20534472	GSM545783	GSE21763	387977	Mov10L1 IP	wild type adult testis
132	20022248	GSM475279	GSE19172	1815424	Miwi IP	adult testis
133	20022248	GSM475280	GSE19172	2625847	Mili IP	adult testis
134	24787618	N/A	N/A	879	Miwi CLIP	elongating spermatids

List of piRNA datasets download from piRBASE (http://www.regulatoryrna.org/database/piRNA/index.html) used for generated de IPpiRNAdb. Dataset identification number in piRBASE (piRBASE ID), ID of the publication of the dataset in PubMed (PubMed), accession numbers of datasets in NCBI Gene Expression Omnibus database (Accession, Series), number of unique sequence in dataset (Uniq Seq), method and target of inmunoprecipitation (Method) and tissue and developmental time used for the inmunoprecipitation (Tissue), were showed in the table.

Supplementary Table 2-5 can be downloaded from GitHub:

https://github.com/edugenetico/Immunoprecipitation-piRNA-database/raw/Supplemental-Tables/Supplemental_Tables2-5.pdf
